# Ageing with HIV: challenges and coping mechanisms of older adults 50 years and above living with HIV in Uganda

**DOI:** 10.1186/s12877-024-04704-z

**Published:** 2024-01-24

**Authors:** Scovia Nalugo Mbalinda, Derrick Amooti Lusota, Martin Muddu, Mathew Nyashanu

**Affiliations:** 1 Department of Nursing, College of Health Sciences, School of Health Sciences, Makerere University, Kampala, P.O Box 7072, Uganda; 2 Makerere University Joint AIDS Program (MJAP), Kampala, Uganda; 3 Department of Health & Allied Professions School of Social Science, Nottingham Trent University, Nottingham, UK

**Keywords:** Experiences, Challenges, Ageing with HIV, Less developed country

## Abstract

**Introduction:**

Globally, adults 50 years and older are an increasing proportion of persons living with HIV (PLHIV), accounting for 16% of the patient group globally. The long-term effects of antiretroviral use are still being discovered and have been associated with several comorbidities; Stigma presents challenges for those in need of services and health care and can significantly affect mental health and treatment adherence. Understanding the experiences and challenges of older PLHIV will inform the development of interventions to improve their care, health, and quality of life, which may help prevent the further spread of HIV. We explored the experiences and challenges of older PLHIV aged 50 years and above.

**Methods:**

We conducted 40 in-depth interviews with elderly PLHIV aged 50 years and above who had lived with HIV for more than ten years. We also explored the experiences and challenges of ageing with HIV in two hospitals. We analysed the data thematically.

**Results:**

The key themes that emerged included; late diagnosis of HIV, depression and fear at the time of diagnosis, acceptance of close family, stigma from community, polypharmacy, development of comorbidities, financial burden, resilience, and mastery of own care.

**Conclusion:**

Older adults experience several challenges, and there is a need to develop special clinics providing appropriate care for the ageing and their social life. Prevention, Early diagnosis and appropriate treatment of HIV, and appropriate geriatric care are essential for the well-being of elderly PLHIV.

## Introduction

Evidence show that some people who were diagnosed with HIV in the 1990s and accessed Antiretroviral Therapy (ART) are now getting older [1]. Long life span while living with HIV is attributed to the availability of antiretroviral Therapy (ART) especially from the year 2000 onwards. Despite these phenomenal breathtaking advances in therapy for HIV infection, ART has limitations for people ageing with HIV. The successful treatment of HIV infection requires consistent daily uptake of ART medication for life among persons living with HIV (PLHIV) [2]. This also requires a robust health delivery system to effectively manage the treatment of HIV infection while dealing with other infections. This complex problem creates a formidable challenge in low and middle-income countries (LMICs), where most patients have no comprehensive health coverage.

Although effective ART enables PLHIV to attain a normal life expectancy like that of the general population, there has been a critical challenge associated with comorbidities and other ailments that come with age which have not been addressed due to poor transition processes [3]. Therefore, an effective and robust transitional care program for individuals crossing from adult to geriatric HIV care is made available within the health care system to enable positive outcomes for people living with HIV (PLHIV) [4, 5]. The current indicators used by the United Nations General Assembly Special Session on HIV/AIDS are more biased towards children and adults younger than 50 years of age [6], while the HIV-related indicators for the then Millennium Development Goals required reporting on those individuals aged 15–24 [7]. There is a need to start tracking older PLHIV, so they are supported effectively through their transitional journey.

It is evident that a substantial proportion of PLHIV are now 50 years and older, estimating that around 2.8 million adults are living with HIV in this age group [8]. This has implications for the changing terrain of HIV care among people aged with HIV [9]. In light of this assertion, there is a need to consider the issue of ageing with HIV as an urgent and important issue if the quality of life among people ageing with HIV is going to be improved. It is also important to note that older age is a significant risk factor for severe and even fatal comorbidities, especially when it is compounded with HIV.

Other opportunities to highlight the emerging trend of the ageing of the HIV epidemic and the potential impact on the HIV response have been missed. For example, in June 2011, the UN convened a high-powered meeting on AIDS in New York, and this was ten years after the historic 2001 United Nations Special Session on HIV/AIDS. The meeting was meant to account for the progress and challenges of the last 30 years and shape the future AIDS response [8]. Despite the clear, forward-looking objective of the meeting, the resolutions failed to acknowledge the ageing of HIV and its implications, mentioning the relationship between the HIV and NCDs epidemics only in the context of a long list of conditions with programmatic overlaps with HIV [10]. AIDS 2031, the group put together by UNAIDS to lead the actions required to robustly address the trajectory of the HIV epidemic over the coming decades, has called for the need to shift the response from “crisis management to sustained strategic response [11]. Despite its crucial mandate in its recent publication, the group has no clear strategy to respond robustly to the formidable challenge of ageing and HIV over the coming decades. Uganda is one of the countries that saw a rise in the number of HIV infections in the late eighties up to the new millennium [11] The robust response by the government of Uganda and acceptance of health messages enabled PLHIV to live longer. This has prompted the question of how to support these people effectively as they age and need more specialised clinical and social support.

Considering the above assertions, this research study aimed to explore the experiences and challenges of older adults 50 years and above living with HIV in a less developed Country.

## Methods

### Study design

This was an exploratory qualitative study.

### Study setting

Participants were recruited from one district hospital and one regional referral public hospital in central Uganda. The health facilities offer a full range of services that include preventive, promotive, curative, maternity, in-patient health services, surgery, blood transfusion, and laboratory and medical imaging services. The facilities also provide HIV services, including HIV testing services (HTS), prevention of mother-to-child transmission (PMTCT), voluntary male medical circumcision (VMMC), provision of antiretroviral therapy (ART) and the management of certain HIV-related comorbidities.

### Sampling strategy

Participants were purposively selected from public health facilities. The participants were 50 years old and had been on ART for ten years and more.

### Participant selection

The researchers recruited 40 participants from August to September 2020. Participants were purposively selected from public health facilities. The participants were 50 years old and had been on ART for ten years and more.

The inclusion criterion was the willingness to participate in the study; participants were 50 years old and had been on ART treatment for ten years and more. Participants gave individual written consent before voluntary participation. The researchers explained the purpose of the study and sought their consent to take part.

### Data collection procedures

Prior to data collection, an in-depth interview guide was pretested among older patients living with HIV in another clinic. Socio-demographic characteristics were obtained from study participants. The study employed semi-structured in-depth interviews (IDIs) to collect data to understand the challenges and experiences from the research participants. The research team conducted IDIs in a private meeting room at the health facility. Each IDI lasted 45 min to 1 h. The lead researcher conducted in-depth interviews and has experience conducting qualitative studies for the last ten years. An interview guide with open-ended and probing questions was used to collect data. Participants decided whether to interview in English or the local language. All interviews were audiotaped. Data collection stopped when no significant new information emerged from interaction with the participants.

### Data management and analysis

Data were transcribed verbatim, and transcripts were returned to participants for comments and corrections. A language expert translated the approved transcripts into English. Two researchers independently coded the data, then identified and highlighted concepts and key phrases and obtained emerging themes. The codes were aggregated into themes (groups of word patterns or phrases with similar meanings) to describe the experience of ageing with HIV. The NVivo 10, a software package, was used to manage the data. Data analysis involved the evaluation of audio recordings and field notes to transcribe the data. The transcripts were analysed by thematic analysis described by Mays and Pope [12]. Data analysis employed the constant comparative method to analyse codes or meaning units (recurrent patterns statements, words or phrases with similar meaning or interpretation) across the data set [13, 14]. Representative quotes from participants derived from the individual transcripts were included to illustrate the source of interpretations of information. The researchers carefully avoided compressing the data too much to keep the findings’ richness and distinctiveness. All text excerpts were de-identified. A sample of participants provided feedback on the findings to enhance the trustworthiness and credibility of the data analysis.

### Ethical consideration

Ethical reviews and approval were obtained from the Research Ethics Committee of the School of Health Sciences, College of Health Sciences at Makerere University (MAKSHSREC-2021-104) and Uganda National Council of Science Technology (HS1759ES). Administrative clearance and permissions were also obtained from the management of each of the health facilities. All participants gave written informed consent to participate. This consent was obtained at the time of their visit to the ART clinic. All participants gave written informed consent to participate. Participants who could not read or write (in English or Luganda) were asked to provide verbal consent after the script was read to them by the interpreter to assist in the comprehension of the research and verbal consent process; those who could read and write signed a written consent form by putting a thumb print. Any information shared about unpleasant experiences was discussed in an empathetic manner. Participation was voluntary, and all the interviews were conducted in private to ensure participant confidentiality. Participants who seemed emotionally affected by the recall of unpleasant experiences were offered a session for counselling. Participants were assured that they were free to participate, decline, and draw from the study anytime. Their decision would not affect the care that they were entitled to. All methods were performed in accordance with the relevant guidelines and regulations.

## Results

The challenges experienced by the research participants included late diagnosis, fear at the time of HIV diagnosis, acceptance from closer family, stigma from the community, development of comorbidities, and financial burden. In coping with the challenges, the research participants reported resilience, mastery of care and hope for the future.

### Participants’ characteristics

Participants were 50 to 71 years old, the majority were married, and all of them had spent ten years or more on ART and acquired HIV through sexual intercourse (Table 1).

**Table 1:** Socio-demographic characteristics of the participants

Variable	Frequency (*N* = 40)	Percentage (%)
**Age**		
50–54	17	42.5
55–59	11	27.5
60–64	8	20
65–69	3	7.5
70–74	1	2.5
**Sex**		
Male	19	47.5
Female	21	52.5
**Marital status**		
Married	20	50.0
Single	2	5.0
Divorced/separated	9	22.5
Widowed	9	22.5
**Religion**		
Catholic	26	65.0
Anglican	9	22.5
Muslim	2	5.0
Born again	3	7.5
**Level of education**		
Illiterate	5	12.5
Primary	18	45.0
Secondary	9	22.5
Tertiary/university	8	20.0
**Employment status**		
Employed	30	75.0
Unemployed	10	25.0
**Number of children**		
0–4	23	57.5
5–9	14	35.0
10–14	2	5.0.0
15–19	1	2.5.0
**Time since HIV diagnosis in years**		
10–14	18	45.0
15–19	18	45.0
20–24	4	10.0
**Time since ART initiation**		
10–14	21	52.5
15–19	17	42.5
20–24	2	5.0
**How were you infected with HIV?**		
Unprotected sex	40	100

### Late diagnosis

Since most of these participants got HIV early in the epidemic, they did not know they had HIV. They observed that they fell sick often and would go to hospitals and clinics and not get better. Most of them knew that they had HIV during these hospital visits

I got sick, and the health workers thought that it might be cancer or diabetes, but they tested for all those diseases, and they were negative. But it developed following a knock…………. Even though they didn’t find those diseases, the health workers decided that to preserve my life, they had to cut off my limb. They first cut it off below the knee, and later when the bone grew, they realised that the way it had grown was not good. Then they cut it the second time, so they cut it twice. After some time, the other one also got infected, and still, they realised that it was also supposed to be cut off to save my life. So right now, I don’t have any toe or a leg. They were cut above o cannot even crawl. By the time I was amputated, I was already tested for HIV and even started treatment. (Male, 63 years)

Other participants had a late diagnosis because of denial and the fear of what would happen after HIV diagnosis:

Someone told us that our husbands whom we have gotten married to had HIV, which I didn’t believe. Time passed, and we left there and came here. It would take some time for one to become critically ill following infection, but someone told us that we had HIV my husband and me because they knew his past relationship……………. My husband started frequently falling sick. So, I would remember the rumour they had told me that we have HIV, but he would deny it when I told him. so we were there like that until when he became critically ill. So we didn’t test until he was critically ill. (female, 58 years).

### Fear at the time of diagnosis

Most of the study participants did not choose to go and test; however, they were encouraged by the health workers at that time to go and test because of their histories of falling sick all the time. Those who had tested positive at that time; experienced a lot of fear and were afraid of the unknown; since most people were dying at that time.

I got so worried. I was like, why me a person who has never cheated on my husband. So, I first hated the man I had at that time because I knew it was him who infected me with HIV. (Female, 58 years)

I went back home so broken and weak! That time I was asking God why he had allowed it to happen because I had prayed for a husband, and He had given me a husband. Now that time, his family had taken my husband, and I could no longer see him (Female, 52 years).

Some of the participants were so worried but were helped by the health providers to be strong

Those results made me get so worried, but counsellors helped me so much! They told me that testing positive does not mean that you are going to die. They told me some things which I had to avoid, for example, having sex with women before testing and told me not to worry so much about what I could not do. (Male, 60 years)

### Acceptance from close family

Most of them said the disclosure was hard and challenging; however, they had to disclose it to their immediate family, and most of the families were sad but accepted and supported them then. Those who had young children waited until they were old enough to disclose their sero status.

Telling them it really took me some time. Except that one of them, actually, the youngest one time asked me why I take that medicine every day………………. At that time, he was in P3; I told him frankly that there is a disease called “Siliimu”. He had heard about it but did not know what it meant; even at school, they were telling them about that disease. He asked me that same question three times on different days until I had to explain to him that it’s what caused her mother to die. Because I knew the moment he had asked me, if I didn’t tell would ask someone else. So I told him but cautioned him never to tell anyone. So even that child became very key in encouraging me to take medicine because I had told him that if one does not take that medicine well, it could lead him to death. So he would encourage me to take medicine every time I could finish having food. So he became so close to me because of that, and even now, when he is giving money, he considers going to the hospital first. He calls you every time it approaches dates of picking my medicine, asking if I have transport and even reminding you.

### Stigma from community

The participants said they faced a lot of stigma from the community, and this affected them for some time. A lot of community education occurred through the health facilities and implementing partners, and some of those affected chose to educate the communities they lived in

Those days things were so hard, you could even leave the place you were renting. For me, it was just the grace of God that helped me, but I think I would produce all these children with the virus. Even if people saw your child, they would think that the child has HIV too! Your child could just get malaria, and they start saying, “the virus has started on her daughter”. Surprisingly, those who were pointing at us, some have even died. They could say, these ones are dying anytime. (Female, 59 Years).

For me, I didn’t get any fear or, as people say, stigma. But I was surprised about the people’s reaction, for them, they took it differently, they didn’t want to engage with me so much, but for me, it could give me freedom telling someone my status, but for some people, they didn’t want to interact with me and socialise with me. (Male, 72 years).

### Development of comorbidities

Some participants have developed comorbidities like diabetes, sexual dysfunction, and joint pains. They attribute this to ageing and the side effects of the drugs they are taking

I came here, and I was tested, and they found that it was severe. I used to sweat a lot and drink plenty of water, then I was like, maybe the drugs I am on are so strong, and they are the ones making me sweat a lot. But when I reached here and tested, they found out that it was severe and I was started on insulin injections. (Female, 58 years)

it was the health workers who told me because I came and told them that I feel as if something isn’t right about my life, and when they checked, they told me that the blood sugar levels were high. And also, for all those many years I had spent on ARVS, I had no diabetes. So, one of the health workers here said that one of the side effects of this drug it causes diabetes and immediately, they changed my regimen and removed DTG. I didn’t spend a lot of time on it. But what is bad is that diabetes still continued even after stopping the DTG. (Male, 70 Years).

### Financial burden

The research participants reported a lack of finance due to being single parents or not being employed following a long episode of illness.

My husband died and left me with four children, I have had to raise them to educate them, but it has not been easy…. I also lost my job following an episode of a long illness. (Female, 58 years).

Initially, the ART drugs were expensive, but now they are free……………. and we are also advised to eat well, but sometimes you can’t afford a good meal because you have many people you are looking after. (Male, 60 Years).

### Resilience

Most of the research participants reported developing tough skin and have seen that the medicine works and they hope and know that they will live for more years like any other person.

I feel like there is something missing because, as someone who was doing well initially and then seeing this happening, it takes some time to accept it and get used to it. (Female, 57 years)

From the time I started septrin up today, I feel so good than before. I feel a lot of energy than before. I can carry out my work better than before. (Female, 68 years)

### Mastery of care and hope for the future

The research participants reported taking ARVs for more than ten years, have accepted this, and now know what to do to improve their quality of life.

Truthfully, I stopped thinking about the virus because I know my life went back to normal like any other person and I do all my things like how any other person would do because I know that I am a normal person but just with the virus and I have learnt to take care of my self. (Female, 65 years)

When I was told that I had HIV, I thought that I could not educate my children from Primary to finish university. But the firstborn completed the course at the university, the second one also completed the course, though it did not go far. The third one, who is a girl, also completed the accountancy course! So, if I am to live, I have now hope in my children! (Male 60 years)

## Discussions

HIV is highly stigmatised in many communities making many people not even ask for an HIV test when sick [15]. Furthermore, many older people view HIV as a condition that can only affect young people again, causing them to make no effort to take an HIV test when they are sick and visit a hospital [16]. In this study, the research participants got HIV early in the epidemic; and did not know about HIV. They observed that they fell sick often and would go to hospitals and clinics and not get better. Most of them knew that they had HIV during these hospital visits. There is a need to normalise HIV testing at treatment centres for all ages so that no one is missed. Through the Ministry of Health, the central government needs to launch a widespread health promotion drive to reduce HIV stigma and encourage older people to test when they visit a health facility.

The stigma associated with HIV is one reason why many people feel so fearful once they get a diagnosis [17–19]. HIV infection has many implications concerning people s’ attitudes towards people living with the condition [20]. There are also issues concerned with disclosure to relatives and others close to the affected person. In this study, most of the participants did not choose to go and test; however, they were encouraged by the health workers at that time to go and test because of their histories of falling sick all the time. Most people who tested positive at that time; experienced a lot of fear and were afraid of the unknown; since many people were dying at that time. More importantly, through the Ministry of Health, the central government should create safeguards in communities for older people PLHIV by establishing emotional supporting services. Community-based initiative hinged on the African ethos of Ubuntu, meaning I am because we should be supported to reduce HIV stigma and increase testing uptake. Such initiatives would caution newly diagnosed people to continue their life normally following an HIV diagnosis.

Disclosure following HIV infection is a very difficult process, especially when the affected person is old and commands respect from the community [21]. However, it is important for affected individuals to disclose their HIV status to close relatives and friends to ensure they are supported effectively. Most research participants recounted that disclosure was hard; however, they had to disclose it to their immediate family. Most of the families were sad but accepted and supported them then. Those who had young children waited until they were old enough before they could disclose their status to them. There is a need for the government, through the ministry of health, to work with communities to understand the importance of supporting older people who disclose their HIV status. Such an initiative will encourage older people to disclose their HIV status without fear of being stigmatised or ridiculed. It is also important that communities know the many ways people can contract HIV and be ready to accept them without judging them.

Stigma from the community following a contraction of HIV is very rife in many neighbourhoods [22]. This can cause stress and discomfort for the individual diagnosed with HIV. In many circumstances, owing to the high rate of HIV stigma in communities, many affected people will choose not to disclose their status and sometimes go as far as stopping taking the medication in the presence of many people [23]. In this study, the research participants said that they faced a lot of stigma from the community, which affected them for some time, but others chose to educate the communities they lived in. There is a need to roll out a nationwide health promotion programme in communities to reduce or prevent the stigmatisation of older PLHIV. In other successful programmes to reduce HIV stigma in communities, PLHIV have been incorporated to educate the community about how stigmatisation impacts their lives [24]. This has been very successful in many circumstances because they have lived experiences of stigmatisation and many people often listen to them.

Ageing with HIV is challenging because of other comorbidities that may come with age [22]. Many older PLHIV tend to present at treatment centres with other associated conditions [25, 26]. This can have a dire impact on the PLHIV as it may increase the amount of medication they will be taking daily, leading to more side effects. The research participants reported developing comorbidities like diabetes, sexual issues, and joint pains. The government needs to have a clear pathway to cater geriatric needs as these people age with HIV through the ministry of health. Such a service will be prepared to care for comorbidities that may eventually affect older people. Furthermore, it is also important that community nurses regularly visit older PLHIV to check their condition and provide ongoing care within their homes. This can provide early opportunities to pick up new infections or comorbidities before people are severely affected.

Many older PLHIV in developing countries have regular financial challenges [27, 28]. This makes it difficult for them to have a good nutritional diet while taking medication. Lack of finance has also prevented PLHIV from attending important hospital appointments [29]. The research participants reported a lack of finance due to being single parents or not being employed following an extended episode of illness. There is a need for the central government to provide support for PLHIV to make sure that their families have enough food. Furthermore, there is a need to create employment opportunities for PLHIV who lost their jobs due to long episodes of sickness. Such opportunities can provide economic reprieve to PLHIV.

In coping with the challenges, the research participants reported resilience, mastery of care and hope for the future. Resilience is a very important virtue for a person living with HIV to navigate, resist and cope with community HIV stigma [30]. The concept of resilience, defined as a dynamic process that allows human beings to overcome adversities, is essential for understanding HIV infection and the treatment of AIDS patients [31]. It helps decrease stigmatisation and prejudice towards the disease and PLHIV. Most of the research participants reported developing tough skin and have seen that the medicine works and they hope and know that they will live for more years like any other person. There is a need for the government, through the ministry of health, to support and sustain this resilience to achieve positive health outcomes for older PLHIV.

Knowing to care for oneself among PLHIV is a very important phenomenon and key to positive health outcomes [32]. Such a skill can also boost the confidence of PLHIV. Adherence, knowledge and belief in the positive working of ARVs can be a sign of hope for the future among PLHIV [33] The research participants reported taking ARVs for more than ten years, have accepted this, and now know what to do to improve their quality of life. There is a need for the government, through the ministry of health, to continue raising awareness of the importance of taking ARVs to sustain positive adherence to medication among older PLHIV.

### Limitations of the study

This research was only conducted in one county in Uganda future similar studies need to be carried out in different counties to enable comparison. The research also used a qualitative approach; this may not be generalised beyond the area where research is conducted and could be noted as a limitation of a qualitative approach. Future mixed methods research may need to be undertaken to examine issues from different epistemological and ontological positions.

### Implications for practice

There is a need to develop a service that will cater for older PLHIV and other comorbidities. The service needs to be staffed with professionals who have good knowledge of geriatrics health and HIV. More conversations are needed with older adults living with HIV to understand the type of service they would need in future.

## Conclusion

Older PLHIV experience many challenges that, include the emergence of comorbidities associated with ageing. There is a need to have a clear pathway to cater for older PLHIV. Furthermore, there is a need for continuous health promotion to reduce HIV stigma and help older people cope with HIV in later years.

## Data Availability

The acquired and/or analysed data are not publicly available because of the lack of authorisation from the agreement with the Research Ethics Committee that the database would remain with the corresponding author only. However, all data can be made available by the corresponding author upon reasonable request.

## Abbreviations

ART: Antiretroviral Therapy
HIV: Human Immunodeficiency Virus
MOH: Ministry of Health
PLHIV: persons living with HIV
